# Spontaneous Renal Artery Dissection in COVID-19 Pneumonia: Potential Danger of Cytokine Storm

**DOI:** 10.1155/2021/6696443

**Published:** 2021-04-10

**Authors:** Jitendra Parmar, Tapan Patel, Sandip Shah, Jay Kothari, Sameer Dani, Sagar Vyas

**Affiliations:** ^1^Department of Radiology, Apollo Hospitals International Limited, Ahmedabad, India; ^2^Department of Intensive and Critical Care, Apollo Hospitals International Limited, Ahmedabad, India; ^3^Department of Interventional Cardiology, Apollo Hospitals International Limited, Ahmedabad, India

## Abstract

The coronavirus disease (COVID-19) pandemic has rapidly spread across the globe since its first detection in March 2020. Its widespread manifestations and vascular complications are increasingly being reported even in young and middle-aged patients. Hyperinflammation is a continuum of host's exaggerated inflammatory response representing cytokine dysregulation/storm which produces coagulopathy and vascular endothelial dysfunction, apart from a prothrombotic state. Cytokine storm or direct viral invasion of the vascular endothelial cells through surface angiotensin-converting enzyme 2 receptors may result in endothelial dysfunction which can potentially result in dissection. Only a few case reports have been published in the literature describing vascular dissection without any inciting factors in COVID-19 patients. Herein, we present the first case report of bilateral renal artery dissection in a 41-year-old male patient who recently recovered from COVID-19 and was managed successfully in stages after many medical hurdles.

## 1. Introduction

Coronavirus disease (COVID-19) was declared as a pandemic in March 2020; since then, it has rapidly spread across the globe and infected well over 54 million people worldwide [1]. Severe disease and complications are predominantly seen in older population with comorbidities. However, its widespread manifestations and vascular complications are increasingly being reported even in young and middle-aged patients [2, 3]. In most of the instances, the respiratory system is affected; however, multiorgan involvement is also well documented [2–4].

The clinical manifestations are primarily classified into three stages: stage I of early infection, stage II of pulmonary involvement, and stage III of hyperinflammation [3]. Hyperinflammation is a continuum of host's exaggerated inflammatory response representing cytokine dysregulation/storm which produces coagulopathy and vascular endothelial dysfunction, apart from a prothrombotic state [5, 6]. COVID-19 produces a severe hypercoagulable state which may lead to both venous and arterial thromboembolic events such as stroke, pulmonary embolism, renal infarcts, and limb ischemia [7–9]. Cytokine storm or direct viral invasion of the vascular endothelial cells through surface angiotensin-converting enzyme 2 (ACE2) receptors may result in endothelial dysfunction which can potentially result in dissection [10, 11].

Only a few case reports have been published in the literature describing vascular dissection without any inciting factors in COVID-19 patients [12–15]. Herein, we present the first case report of renal artery dissection in 41-year-old male patient who recently recovered from COVID-19.

## 2. Case Report

### 2.1. 1^st^ Instance

A 41-year-old middle-aged male patient, a healthcare physician by profession, presented with a history of fever and cough for 5 days in late August 2020. As he had a close contact history with a COVID-19 pneumonia patient, he got tested for COVID-19 pneumonia and was found positive. Hence, he was isolated in home quarantine and was started with supportive medications (antipyretic for fever, good hydration, multivitamin tablets, zinc supplements, breathing exercise, salt water gargles (three times a day), and steam inhalation (four times per day)). His symptoms improved significantly, and recovery was uneventful in the next few days.

### 2.2. 2^nd^ Instance

In late September 2020, he again presented with sudden onset severe left flank pain accompanied by fever and nausea. He was febrile with mild tachycardia and oxygen saturation of 97%. The blood investigations revealed raised inflammatory markers, including total leucocyte counts (TLC) (16912/cumm) (4000–11000/cumm), C-reactive protein (CRP) (68 mg/L) (0.00–5.00 mg/L), interleukin-6 (395 pg/mL) (less than 7 pg/mL), lactate dehydrogenase (1569 U/L) (135–225 U/L), serum glutamic-pyruvic transaminase (SGPT) (186 U/L) (less than 45 U/L), serum glutamic-oxaloacetic transaminase (SGOT) (127 U/L) (less than 40 U/L), and D-dimer (1.74 *μ*g/mL FEU) (0.00–0.5 *μ*g/mL FEU). Serum creatinine was 1.2 mg/dL (0.6–1.3 mg/dL). The activated partial thromboplastin time (aPTT) was high (83.4 sec). Initially, he was diagnosed as a persistent or recurrence of COVID-19 pneumonia with moderate illness, hence investigated and treated according to institutional protocol for COVID-19. He was started on antipyretics; low-dose steroids; antibiotics; low-molecular-weight heparin (LMWH) (enoxaparin sodium injection) (40 mg/0.4 mL), subcutaneously once daily; analgesic; antiemetic; antacid; intravenous fluids; and other supportive care. His vitals were closely monitored and regularly investigated for TLC, D-dimer, and CRP. There was no past history of previous surgery, trauma, renal artery intervention, hypertension, cardiac pathology, thrombotic events, or dissection. Electrocardiography and echocardiogram were normal.

**Figure 1:**
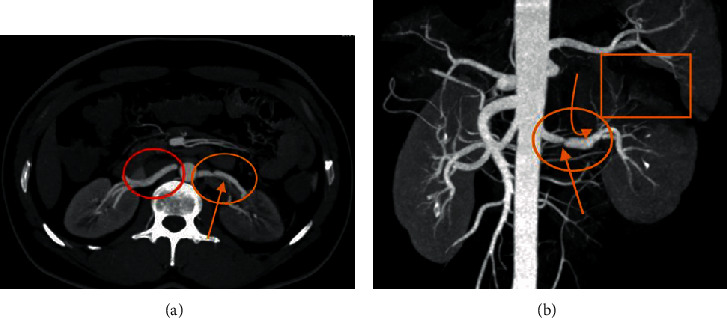
(a) Axial MIP CT image and (b) anterior volume-rendered 3D MPR coronal image were obtained after intravenous contrast material in arterial phase demonstrates focal thrombosis in the mid-left main renal artery (orange circle) and dissection flap (straight orange arrow in a), causing significant stenosis (orange straight arrow in b) and near-complete nonopacification of the upper segmental branch of the left renal artery for a length of approximately 3–4 mm with narrowed caliber and attenuated flow in the rest of the segmental and subsegmental branches (angled orange arrow in b), resulting in patchy cortical nonenhancement, predominantly involving the upper half and few discrete areas in the lower half of the left kidney, suggestive of infarct (orange rectangular in b), and also demonstrates normal right renal artery (red circle in a).

Because of persistent abdominal pain despite the graded analgesia, contrast enhanced CT (CECT) of abdomen was performed (Figure 1). It revealed focal thrombosis in the mid-left main renal artery, causing significant stenosis. There was associated near-complete nonopacification of the upper segmental branch in the left renal artery with narrowed caliber and attenuated flow in the rest of the segmental and subsegmental branches, and eccentric thrombosis with beaded appearance of the middle segmental branch, causing approximately 90% stenosis with attenuated flow in its subsegmental branches. Patchy cortical nonenhancement was seen involving the upper half and few discrete areas in the lower half of the left kidney with mild surrounding fat stranding and thickening of perinephric fascia, suggestive of infarct. At this instance, the right renal artery, right kidney, and superior mesenteric arteries were normal. The findings were initially thought to be associated with recanalized thrombus of distal left main renal artery; however, on retrospective review, a dissection flap was evident. The options of medical management with anticoagulants or endovascular intervention were considered and discussed with the patient. As the patient was hemodynamically stable with no hypertension, pseudoaneurysm, or other comorbidities and normal serum creatinine, medical management was preferred. He was started on heparin infusion and dose adjusted as per aPTT monitoring. He improved symptomatically and was continued on heparin infusion and other supportive medical management. Once aPTT was found to be satisfactory, he was switched over to LMWH. He was discharged in stable condition after 6-7 days of admission on antiplatelet (aspirin (75 mg), once a day) and anticoagulant (enoxaparin sodium injection (60 mg/0.6 mL), subcutaneously, twice daily) treatment.

### 2.3. 3^rd^ Instance

**Figure 2:**
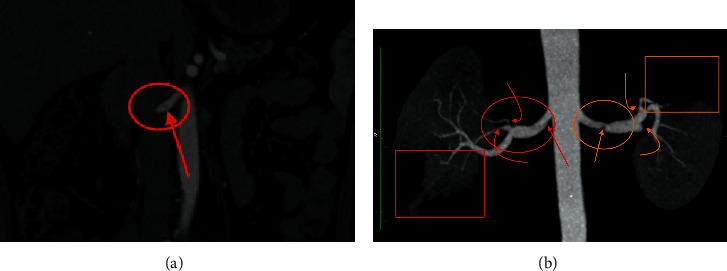
(a) Coronal CT angiography image and (b) anterior volume-rendered 3D MPR coronal image were obtained after intravenous contrast material in arterial phase reveals dissection flap (straight red arrow) of the right renal artery (red circle) in (a); (b) demonstrates focal eccentric thrombosis and attenuated flow and reduced caliber of the lower lobe segmental branch (curved red arrow) and upper lobe subsegmental branch (angled red arrow), resulting in infarct (red rectangular); and also demonstrates focal thrombosis in the mid-left main renal artery and dissection flap (orange circle), causing significant stenosis (orange straight arrow) and complete thrombosis of the middle segmental branch of the left renal artery (curved orange arrow) and partial stenosis of the upper segmental branch (angled orange arrow) with new development of infarct in some part of the mid pole as well (orange rectangular).

After almost 10 days of discharge, he again presented with sudden onset of severe right-sided flank pain, while he had left-sided pain in the previous hospitalization. The blood investigations revealed raised serum creatinine (1.63 mg/dL) and inflammatory markers, including CRP (71 mg/L), plasma fibrinogen (470 mg%) (180–350 mg%), lactate dehydrogenase (653), SGPT (71 U/L), and gamma glutamyl transpeptidase transaminase (83 U/L) (10–71 U/L). D-dimer was 0.31 *μ*g/mL FEU. Coagulation profile evaluation revealed high aPTT (59.2 seconds), high platelet counts (550,000/cumm), and normal prothrombin time (15 seconds) with INR of 1.08. Immunoglobulin G antibody for COVID-19 was positive. CECT abdomen (Figure 2) revealed dissection of the right renal artery with focal eccentric thrombosis and infarct of the lower pole and part of the upper pole of the right kidney. The findings on the left side were almost similar to the previous CECT except the complete thrombosis of the middle segmental branch of the left renal artery and partial recanalization of the upper segmental branch with new development of infarct in some part of the mid pole as well. Extended thrombosis profile and coagulation profile were almost within normal limits, except positive activated protein C resistance, high protein S (free), and moderately positive lupus anticoagulant. Connective tissue disorder workup (cytoplasmic antineutrophil cytoplasmic antibodies (C-ANCA), perinuclear antineutrophil cytoplasmic antibodies (P-ANCA), antinuclear antibody profile, and anti-double-stranded DNA) was within normal limits. Electrocardiogram and echocardiogram were normal at this instance as well. Now at this instance, the endovascular management was considered over medical management and was discussed with the patient.

### 2.4. Therapeutic Intervention

**Figure 3:**
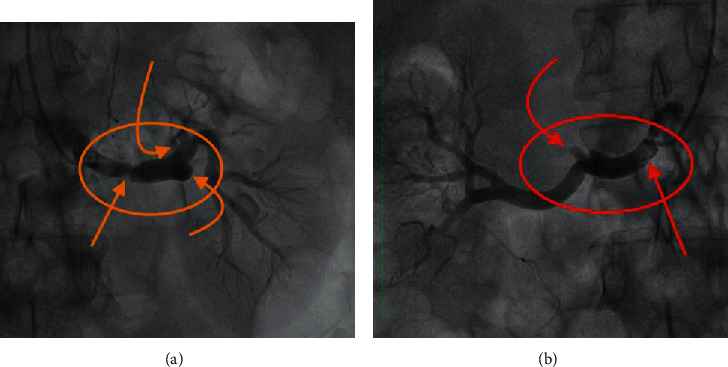
Conventional catheter selective angiography of the left renal artery (a) reveals overall reduced perfusion in the upper half of the left kidney with a focal thrombosis in the mid-left main renal artery (orange circle) and dissection flap, causing significant stenosis (orange straight arrow); demonstrates complete thrombosis of the middle segmental branch of the left renal artery (orange curved arrow) and near-complete stenosis of the upper segmental branch (orange angled arrow); and (b) reveals reduced perfusion in the lower 1/3^rd^ of the right kidney with focal expansion of the right renal artery (red circle) with dissection flap (red straight arrow) and with near-complete stenosis of the segmental branch (red curved arrow) supplying the lower pole and part of the upper pole.

Conventional catheter angiography was performed (Figure 3), which confirmed the CECT findings, and endovascular management was performed sequentially. In the first stage, intra-arterial thrombolysis was performed using urokinase (1.5 lakh bolus dose followed by 1 lakh/hour for the next 24 hours). The perfusion significantly improved in both kidneys on check angiography done the next day after 24 hours of intra-arterial thrombolysis. In the second stage, the balloon angioplasty was carried out after 48 hours of intra-arterial thrombolysis for the mid renal artery and upper segmental artery on the left side and lower segmental and upper segmental artery on the right side. In addition, thrombus reduction technique was also performed by thrombosuction catheter which suctioned jelly-like material.

### 2.5. Final Outcome and Follow-Up

The perfusion of both kidneys improved significantly after endovascular intervention (Supplementary file) and serum creatinine returned to normal. Unfortunately, he developed pseudoaneurysm at femoral artery puncture site which was successfully managed by few rounds of ultrasound-guided compression. During the tight compression, he developed deep venous thrombosis in the right iliac veins, which was successfully managed by anticoagulant therapy. Finally, he was discharged in stable condition after 23 days of admission on antiplatelet, anticoagulant, statin, and low antihypertensive support.

## 3. Discussion

COVID-19 is a well-established pathogen responsible for a spectrum of clinical manifestations, from a simple uncomplicated infection involving the lungs and upper airway tract to a complicated infection including multiorgan dysfunction and varying degrees of vascular involvement. The pathophysiological effects of COVID-19 infection on renal function are not clearly determined, and more studies are underway to understand the pathophysiology [8]. However, published literature and reports have postulated that it causes severe hypercoagulable stage and cytokine storm that may lead to thromboembolic events and vascular endothelial injury that may affect the vessel wall which rarely can lead to dissection [12–15].

We herein presented a rare case of renal artery dissection and thrombosis, leading to infarct in both kidneys. The patient presented in this report had no history of trauma, renal arterial intervention, or connective tissue disorder. Patient denied any history of genetic or hereditary disease. There was no obvious findings on CT or conventional angiography to suggest fibromuscular dysplasia. The inflammatory markers were raised even after 1 month of COVID-19 that suggest the patient was in hyperinflammation stage. The aggravated inflammatory response (cytokine storm) may have led to dissection and thrombosis in the left renal artery, resulting in infarct in part of the left kidney. Even after optimum and appropriate medical management, he developed dissection in the right renal artery as well and infarct in part of the right kidney.

Hence, in view of the absence of other predisposing factors, normal thrombophilia profile and connective tissue disorder workup, in association with the temporal sequence of events, this isolated case in our experience poses the question of a potential association between COVID-19 and renal artery dissection. However, further studies are needed to determine the potential of COVID-19 to cause vascular dysfunction leading to dissection and also if long-term anticoagulation may be used as a prophylaxis in patients with long-standing hyperinflammation. Since this infection is under study worldwide, we suggest that physicians looking after these patients should be aware of this potential cause of vascular dissection.

## Abbreviations

COVID-19: Coronavirus disease 2019
ACE2: Angiotensin-converting enzyme 2
TLC: Total leucocyte counts
CRP: C-reactive protein
SGPT: Serum glutamic-pyruvic transaminase
SGOT: Serum glutamic-oxaloacetic transaminase
LMWH: Low-molecular-weight heparin
CECT: Contrast enhanced computed tomography
aPTT: Activated partial thromboplastin time.

## Additional Points

*Learning Points*. (1) COVID-19 produces severe hypercoagulable state which may lead to both venous and arterial thromboembolic events such as stroke, pulmonary embolism, renal infarcts, and limb ischemia. (2) Cytokine storm or direct viral invasion of the vascular endothelial cells through surface angiotensin-converting enzyme 2 receptors may result in endothelial dysfunction which can potentially result in dissection. *Summary Statement*. With the gradual recognition of clinical-pathological features of COVID-19 pneumonia, it is important to be aware of the spectrum of the clinical and imaging manifestations. Though COVID-19 as an etiology or merely a coincidence with vascular dissection is a matter of debate, its potential as a cause should be kept in mind for rapid and correct diagnosis and treatment, which may contribute to effective surveillance and management.

## Supplementary Materials

Supplementary Materials Supplementary Figure 1: 3D VR image obtained after intravenous contrast material in arterial phase in a 41-year-old male patient who recently recovered from COVID-19 pneumonia with persistent high inflammatory markers presented with sudden onset severe left flank pain demonstrates focal thrombosis in the mid-left main renal artery (orange circle) and dissection flap causing significant stenosis (straight orange arrow) and also demonstrates near-complete nonopacification of the upper segmental branch of the left renal artery for a length of approximately 3–4 mm with narrowed caliber and attenuated flow in the rest of the segmental and subsegmental branches (angled orange arrow) and patchy cortical nonenhancement, predominantly involving the upper half and few discrete areas in the lower half of the left kidney, suggestive of infarct (orange rectangular). Supplementary Figure 2: anterior volume-rendered 3D MPR coronal image obtained after intravenous contrast material in arterial phase in a 41-year-old male patient who recently recovered from COVID-19 pneumonia. Supplementary Figure 3: 3D VR image obtained after intravenous contrast material in arterial phase in a 41-year-old male patient who recently recovered from COVID-19 pneumonia with persistent high inflammatory markers presented with sudden onset severe right-sided flank pain reveals focal expansion with dissection flap (straight red arrow) of the right renal artery (red circle); demonstrates focal stenosis and attenuated flow and reduced caliber of the lower lobe segmental branch (curved red arrow) and upper lobe subsegmental branch (angled red arrow), resulting in infarct (red rectangular); and also demonstrates focal thrombosis in the mid-left main renal artery and dissection flap (orange circle), causing significant stenosis (orange straight arrow) and complete thrombosis of the middle segmental branch of the left renal artery (curved orange arrow) and partial stenosis of the upper segmental branch (angled orange arrow) with new development of infarct in some part of the mid pole as well (orange rectangular). Supplementary Figure 4: power Doppler evaluation of both kidneys for the evaluation of perfusion before ((a) left kidney and (c) right kidney) and after endovascular intervention, revealing significant increased perfusion in both kidneys after endovascular intervention ((b) left kidney and (d) right kidney).
