# Supplementary material for: Spontaneous Renal Artery Dissection in COVID-19 Pneumonia: Potential Danger of Cytokine Storm
Source: Case Rep Crit Care. 2021 Apr 10;2021:6696443. doi: 10.1155/2021/6696443 (PMC8051521; doi:10.1155/2021/6696443)
Supplement: Supplementary Materials — Supplementary Figure 1: 3D VR image obtained after intravenous contrast material in arterial phase in a 41-year-old male patient who recently recovered from COVID-19 pneumonia with persistent high inflammatory markers presented with sudden onset severe left flank pain demonstrates focal thrombosis in the mid-left main renal artery (orange circle) and dissection flap causing significant stenosis (straight orange arrow) and also demonstrates near-complete nonopacification of the upper segmental branch of the left renal artery for a length of approximately 3–4 mm with narrowed caliber and attenuated flow in the rest of the segmental and subsegmental branches (angled orange arrow) and patchy cortical nonenhancement, predominantly involving the upper half and few discrete areas in the lower half of the left kidney, suggestive of infarct (orange rectangular). Supplementary Figure 2: anterior volume-rendered 3D MPR coronal image obtained after intravenous contrast material in arterial phase in a 41-year-old male patient who recently recovered from COVID-19 pneumonia. Supplementary Figure 3: 3D VR image obtained after intravenous contrast material in arterial phase in a 41-year-old male patient who recently recovered from COVID-19 pneumonia with persistent high inflammatory markers presented with sudden onset severe right-sided flank pain reveals focal expansion with dissection flap (straight red arrow) of the right renal artery (red circle); demonstrates focal stenosis and attenuated flow and reduced caliber of the lower lobe segmental branch (curved red arrow) and upper lobe subsegmental branch (angled red arrow), resulting in infarct (red rectangular); and also demonstrates focal thrombosis in the mid-left main renal artery and dissection flap (orange circle), causing significant stenosis (orange straight arrow) and complete thrombosis of the middle segmental branch of the left renal artery (curved orange arrow) and partial stenosis of the upper segmental branch (angled [file 6696443.f1.docx]

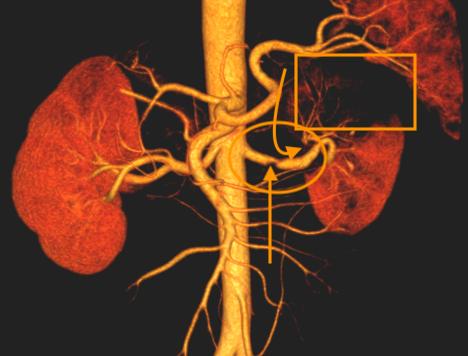


Supplementary Figure 1: -

3D VR image and obtained after intravenous contrast material in arterial phase in a 41-years old male patient who recently recovered from COVID-19 pneumonia with persistent high inflammatory markers presented with sudden onset severe left flank pain, demonstrates focal thrombosis in mid left main renal artery (orange circle) and dissection flap causing significant stenosis in (straight orange arrow). It also demonstrates near complete non-opacification of upper segmental branch of left renal artery for a length of approximately 3 – 4 mm with narrowed caliber and attenuated flow in rest of segmental & subsegmental branches (angled orange arrow) and patchy cortical non-enhancement, predominantly involving upper half and few discrete areas in lower half of left kidney, suggestive of infarct (orange rectangular).


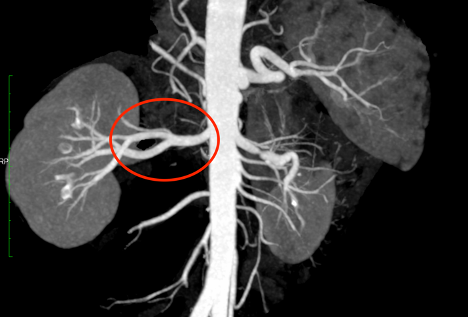


Supplementary Figure 2: -

Anterior volume rendered 3D MPR coronal image obtained after intravenous contrast material in arterial phase in a 41-years old male patient who recently recovered from COVID-19 pneumonia with persistent high inflammatory markers presented with sudden onset severe left flank pain demonstrates normal right renal artery (red circle).


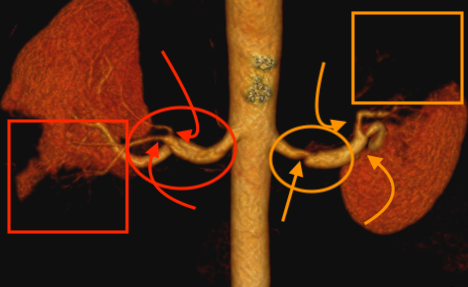
 Supplementary Figure 3: -

3D VR image obtained after intravenous contrast material in arterial phase in a 41-years old male patient who recently recovered from COVID-19 pneumonia with persistent high inflammatory markers presented with sudden onset severe right sided flank pain. It reveals focal expansion with dissection flap (straight red arrow) of right renal artery (red circle). It demonstrates focal stenosis and attenuated flow & reduced caliber of lower lobe segmental branch (curved red arrow) and upper lobe subsegmental branch (angled red arrow), resulting in infarct (red rectangular). It also demonstrates focal thrombosis in mid left main renal artery and dissection flap (orange circle), causing significant stenosis (orange straight arrow) and complete thrombosis of middle segmental branch of left renal artery (curved orange arrow) and partial stenosis of upper segmental branch (angled orange arrow) with new development of infarct in some part of mid pole as well (orange rectangular).


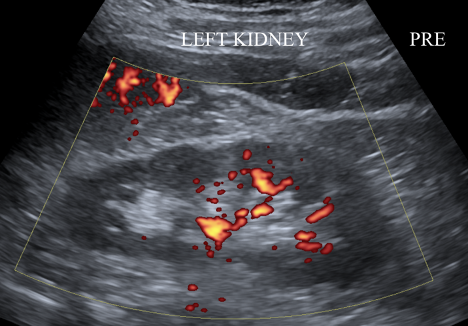


Supplementary Figure 4a: - Power doppler evaluation of both kidneys for evaluation of perfusion before (a – left kidney and c – right kidney) and after endovascular intervention, reveal significant increased perfusion in both kidneys after endovascular intervention (b – left kidney and d – right kidney).

Figure 4b


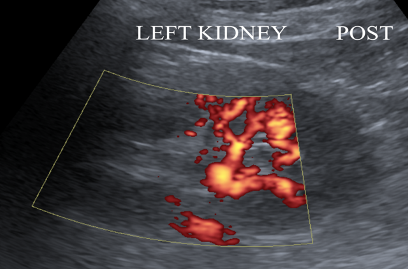


Supplementary Figure 4b: - Power doppler evaluation of both kidneys for evaluation of perfusion before (a – left kidney and c – right kidney) and after endovascular intervention, reveal significant increased perfusion in both kidneys after endovascular intervention (b – left kidney and d – right kidney).

Figure 4c


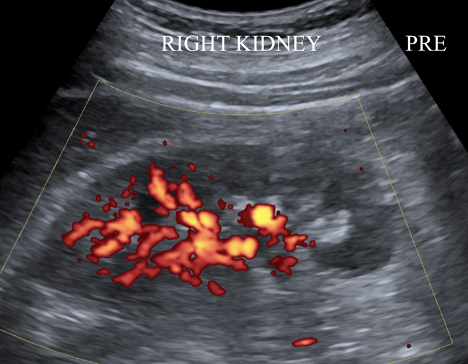


Supplementary Figure 4c: - Power doppler evaluation of both kidneys for evaluation of perfusion before (a – left kidney and c – right kidney) and after endovascular intervention, reveal significant increased perfusion in both kidneys after endovascular intervention (b – left kidney and d – right kidney).

Figure 4d


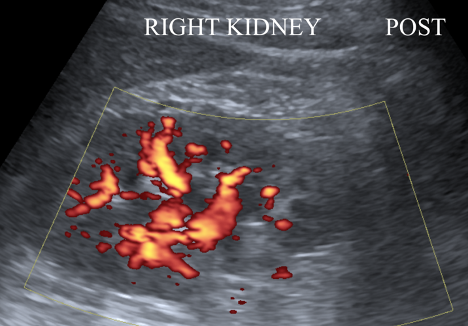


Supplementary Figure 4d: - Power doppler evaluation of both kidneys for evaluation of perfusion before (a – left kidney and c – right kidney) and after endovascular intervention, reveal significant increased perfusion in both kidneys after endovascular intervention (b – left kidney and d – right kidney).
